# Selective digestive decontamination solution used as “lock therapy” prevents and eradicates bacterial biofilm in an in vitro bench-top model

**DOI:** 10.1186/s12941-020-00387-7

**Published:** 2020-09-23

**Authors:** María Jesús Pérez-Granda, Beatriz Alonso, Ricardo Zavala, María Consuelo Latorre, Javier Hortal, Rafael Samaniego, Emilio Bouza, Patricia Muñoz, María Guembe

**Affiliations:** 1 Cardiac Surgery Postoperative Care Unit, Hospital General Universitario Gregorio Marañón, Madrid, 28007 Spain; 2 Instituto de Investigación Sanitaria Gregorio Marañón, Madrid, 28009 Spain; 3 CIBER Enfermedades Respiratorias-CIBERES, CB06/06/0058), Madrid, Spain; 4 Department of Clinical Microbiology and Infectious Diseases, Hospital General Universitario Gregorio Marañón, Madrid, 28007 Spain; 5 Biology Department, School of Biology, Universidad Complutense de Madrid, Madrid, 28040 Spain; 6 Confocal Laser Scanning Microscopy Unit, Hospital General Universitario Gregorio Marañón, Madrid, 28007 Spain; 7 Medicine Department, School of Medicine, Universidad Complutense de Madrid, Madrid, 28040 Spain; 8 Servicio de Microbiología Clínica y Enfermedades Infecciosas, Instituto de Investigación Sanitaria Gregorio Marañón, Hospital General Universitario “Gregorio Marañón”, C/. Dr. Esquerdo, 46, Madrid, 28007 Spain

**Keywords:** Ventilator associated pneumonia, Biofilm, Endotracheal tube, Selective decontamination solution, Lock therapy

## Abstract

**Background:**

Most preventing measures for reducing ventilator-associated pneumonia (VAP) are based mainly on the decolonization of the internal surface of the endotracheal tubes (ETTs). However, it has been demonstrated that bacterial biofilm can also be formed on the external surface of ETTs. Our objective was to test in vitro the efficacy of selective digestive decontamination solution (SDDs) onto ETT to prevent biofilm formation and eradicate preformed biofilms of three different microorganisms of VAP.

**Methods:**

We used an in vitro model in which we applied, at the subglottic space of ETT, biofilms of either *P. aeruginosa* ATCC 15442, or *E. coli* ATCC 25922, or *S. aureus* ATCC 29213, and the SDDs at the same time (prophylaxis) or after 72 h of biofilm forming (treatment). ETT were incubated during 5 days with a regimen of 2 h-locks. ETT fragments were analyzed by sonication and confocal laser scanning microscopy to calculate the percentage reduction of cfu and viable cells, respectively.

**Results:**

Median (IQR) percentage reduction of live cells and cfu/ml counts after treatment were, respectively, 53.2% (39.4%—64.1%) and 100% (100%–100.0%) for *P. aeruginosa,* and 67.9% (46.7%–78.7%) and 100% (100%–100.0%) for *E. coli*. *S. aureus* presented a complete eradication by both methods. After prophylaxis, there were absence of live cells and cfu/ml counts for all microorganisms.

**Conclusions:**

SDDs used as “lock therapy” in the subglottic space is a promising prophylactic approach that could be used in combination with the oro-digestive decontamination procedure in the prevention of VAP.

## Background

Ventilator-associated pneumonia (VAP) is one of the most common pulmonary nosocomial infections in intensive care units (ICU), not only in developed but also in developing countries, with an incidence of 9% to 27% in intubated patients increasing to 46% in patients who need mechanical ventilation for more than 48 h after major heart surgery [1–5]. VAP represents high rates of morbidity and mortality, longer hospital stays, and additional sanitary costs [6].

Although pulmonary aspiration of oropharyngeal and gastric microorganisms are the endogenous rout of airway colonization, the ability of bacteria to form biofilm on endotracheal tube (ETT) surface is thought to be one of the most important external risk factors of VAP development [6–10]. There are several strategies to prevent and treat VAP focused mainly in the internal surface of the ETT, including: selective digestive and oral decontamination (SDD/SOD), use of oral antiseptics, subglottic aspiration, elevation of the head of bed, ETT surface modifications such as antimicrobial-drug coated tubes or silver-coated ETT, mucus removal, and parenteral antimicrobial therapy [9, 11–19].

SDD refers to a prophylactic strategy based on the application of non-absorbable antimicrobial agents in the oropharynx and gastrointestinal tract [20–22]. SDD solution (SDDs) is mainly composed of tobramycin, polymyxins and amphotericin B with antimicrobial activity against gram negative microorganisms, including multi-drug resistant, gram positive, and yeasts; which are the most common causative microorganisms in VAP [1, 20, 22–25]. SDDs was designed to attack only aerobic bacteria potentially pathogenic in the oral cavity and in the digestive tract leaving anaerobic normal microbiota undisturbed [26]. Although there are several studies regarding the efficacy and safety of SDDs in ICU patients [20, 27, 28] literature about its efficacy as antibiotic lock therapy (ALT) or antibiotic lock prophylaxis (ALP) is scarce to the best of our knowledge [29].

Thus, our objective was to assess the efficacy of SDDs as prophylaxis and treatment of bacterial biofilm administered in the external surface of the subglottic space using an in vitro bench model [30].

## Methods

This study was carried out in the laboratory of the Clinical Microbiology and Infectious Diseases Department, Hospital Gregorio Marañón, Madrid, Spain.

Confocal laser scanning microscopy (CLSM) images were performed in the CLSM unit of the Instituto de Investigación Sanitaria Gregorio Marañón. Scanning electron microscopy (SEM) images were performed in the Centro Nacional de Microscopía de Barrido de la Universidad Complutense de Madrid.

### Therapies

**Fig. 1:**
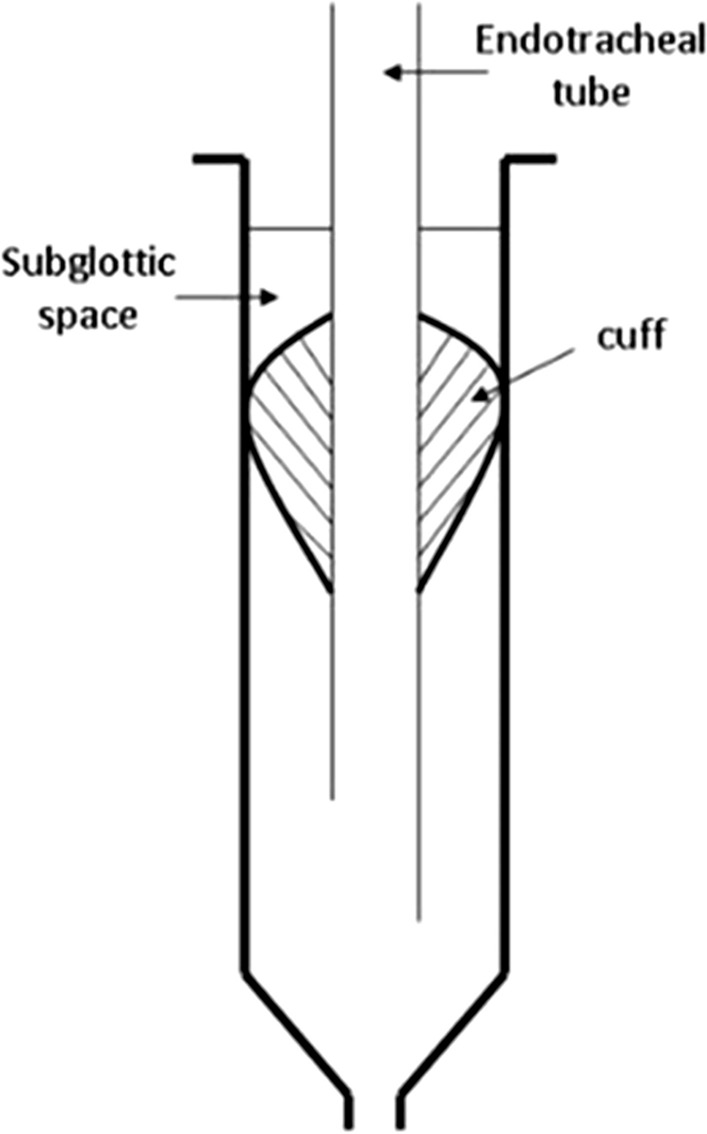
Schematic diagram of the in vitro bench model. A total volume of 3 ml was instilled in the subglottic space for treatment and prophylaxis therapy

An adult tracheal intubation was simulated using cuffed ETT (TaperGuard Oral Tracheal Tube Evac Murphy Eye, Mallinckrodt ™) as shown in Fig. 1 [31].

#### Treatment therapy

*Mature-biofilms*: The ETTs were colonized with 3 ml of 0.5 McFarland culture of *Pseudomonas aeruginosa* ATCC 15442, or *Escherichia coli* ATCC 25922, or *Staphylococcus aureus* ATCC 29213 in their culture medium (BHI, LB, and TSB respectively) (Sigma-aldrich, Spain). ETT were culture at 37 °C for 72 h with 24-h medium replacement performed daily at the same time (Additional file 1: Fig. S1).

*Lock therapy*: ALT was based on a 2-hour application at the mature biofilms of 3 ml in the subglottic area of either SDDs (nystatin 2.6 mIU, tobramycin 15.6 mg/ml, and colimycin 13 mg/ml. In the case of *S. aureus,* vancomycin 3.6 mg/ml was also added) after a washing with sterile saline in the treated samples or sterile saline (0.9% NaCl) in positive controls. Then, solutions were removed and ETT were washed with sterile saline and incubated with fresh medium at 37 °C for 22 h. Antibiotic lock solution was repeated during 5 days. Medium without microorganism was used as negative control. All samples were tested six times.

#### Prophylactic therapy

The ETTs were colonized simultaneously with 1.5 ml of a 0.5 McFarland culture of *P. aeruginosa* ATCC 15442, or *E.coli* ATCC 25922, or *S. aureus* ATCC 29213 in their culture medium (BHI, LB, and TSB respectively) and 1.5 ml of either SDDs in the treated samples or sterile saline (0.9% NaCl) in positive controls. In this ALP, final concentrations of nystatin, tobramycin, colimycin, and vancomycin were as follow: 1.3 mIU, 7.8 mg/ml, 6.5 mg/ml, and 1.8 mg/ml, respectively.

ETTs were incubated at 37 °C for 2 h. Solutions were discarded and ETT were washed with sterile saline and incubated in fresh medium for 22 h at 37 °C. The procedure was repeated during 5 days. Medium without microorganism was used as negative control. All samples were tested six times.

After each therapy, ETT were washed with sterile saline before analysis.

### ETTs analysis

**Fig. 2:**
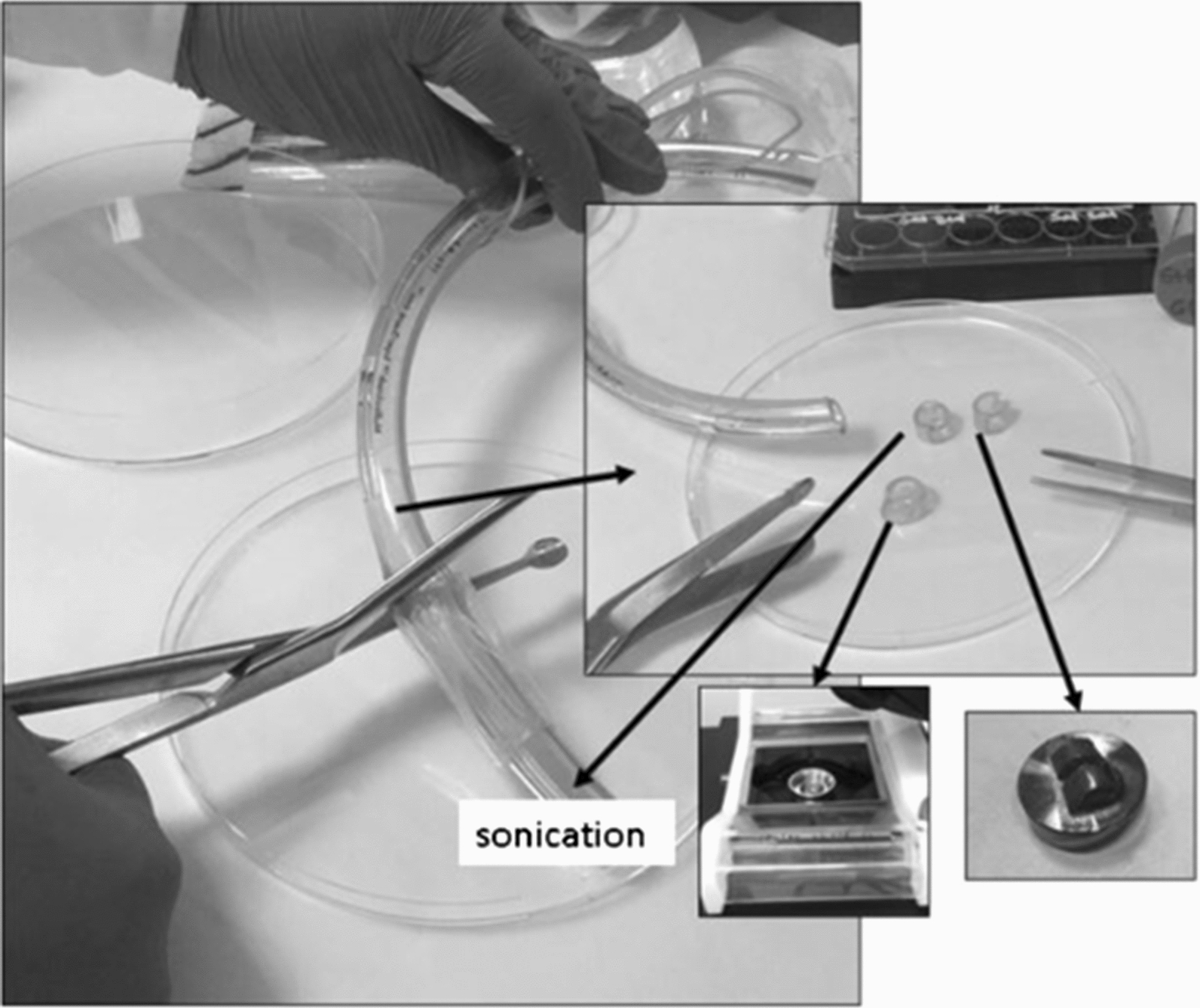
ETT analysis

ETTs were cut into 3 segments of 0.5 cm (Fig. 2). Each segment was used for a different analysis.

*Colony forming unit counts and percentage of live cells*. One segment was sonicated in 2 ml of buffer solution for 1 min at 50 Hz and vigorously vortexed. Solution was then serially diluted and 100 µl were cultured on blood agar plates and incubated for 24 h at 37 °C. We scrubbed ETT surface of positive controls with a sterile swab and was introduced in 1 ml of PBS and 100 µl of the solution were plated on agar plates and incubated for 24 h at 37 °C.

Colony counts were expressed as the number of cfu/ml with a limit of detection of < 10 cfu/ml. Live/dead analysis was performed by centrifuging the remaining sonicate to study the viability of bacteria. Pellet was resuspended in 50 µl of sterile saline and stained with Live/Dead^®^ BacLight kit™ (0.5 µl of SYTO^®^ 9, stock 3.34 mM in DMSO; and 0.5 µl propidium iodide, stock 20 mM in DMSO) (BacLight kit™; Invitrogen, Barcelona, Spain) for 15 min protected from light. A drop (5 µl) of each dilution was mounted on a coverslip and visualized using a confocal laser scanning microscopy (CLSM) in an inverted confocal fluorescence microscope (SPE, Leica Microsystems) equipped with ACS APO 10x/0.30 and ACS APO 63X/1.30 objectives. Images were taken using an ACS APO 63X/1.30 objective. Three images containing over 1000 cells per condition were taken from each sample. Quantification of live and dead cells was performed by using FIJI software (National Institute of Health, US). The percentage of live bacteria was calculated as the ratio between the number of live cells and the total number of cells × 100.

*Visualization of biofilm biomass.* Another segment was fixed by freezing at −80 °C for 72 h. After thawing for 30 min at room temperature, segments were stained with Live/Dead^®^ BacLight kit™ (1.5 µl of SYTO^®^ 9, stock 3.34 mM in DMSO; and 1.5 µl propidium iodide, stock 20 mM in DMSO in 1 ml of buffer solution) for 15 min protected from light [32]. Samples were visualized using CLSM at ACS APO 10X/0.3 objective. Images were edited using FIJI software (National Institute of Health, US).

*Visualization of biofilm structure.* The last segment was used to visualize biofilm structure by scanning electron microscopy (SEM). Segments were placed into 2% glutaraldehyde for 3 days and after dehydration in graded alcohol, samples were sputter-coated with gold atoms. The structure of the treated and non-treated biofilms was visualized using a scanning electron microscope (JEOL-JSM 6400; Jeol, Tokyo, Japan).

### Filtration testing

In order to assess whether “lock therapy” in ETT could leak past the cuff, our bench model was hold into a Falcon tube to recover any filtration of the SDD solution during the therapies.

### Statistical analysis

Qualitative variables appear with their frequency distribution. Quantitative variables are expressed as the median and interquartile range (IQR). Non-normally distributed continuous variables were compared using the Kruskal–Wallis and Mann–Whitney tests.

All statistical tests were 2-tailed. Statistical significance was set at p < 0.05 for all the tests. The statistical analysis was performed with IBM SPSS Statistics 21.0 for Windows (IBM, New York).

## Results

### Overall data

Overall data of the median (IQR) percentage and median (IQR) percentage reduction of live cells and log_10_ cfu/ml for ALT and ALP therapies for *P. aeruginosa*, *E. coli,* and *S. aureus* are shown in Table 1.

**Table 1:** Overall data of live cells, cfu/ml counts, and percentage of reduction of bacterial biofilms after prophylaxis and treatment with selective digestive decontamination solution

Therapy	MO	Median (IQR) % live cells		Median (IQR) % reduction of live cells	P value	Median (IQR) log_10_ cfu/ml*		Median (IQR) % reduction** of cfu/ml*	P value
		C+	ALT			C+	ALT		
Treatment	*P. aeruginosa*	88.9 (84.4–93.4)	39.3 (30.1–50.9)	53.2 (39.4–64.1)	< 0.001	7.5 (7.4– ^b^)	-^a^	100.0 (100.0–100.0)	0.002
	*E. coli*	55.9 (49.6–67.3)	18.8 (12.5–31.2)	67.9 (46.7–78.7)	0.002	7.0 (6.7–7.4)	-^a^	100.0 (100.0–100.0)	0.002
	*S. aureus*	42.9 (28.7–57.5)	0.0 (0.0–0.0)	100.0 (100.0–100.0)	0.007	7.7 (7.5–7.9)	-^a^	100.0 (100.0–100.0)	0.002
Prophylaxis	*P. aeruginosa*	83.1 (77–88.4)	0.0 (0.0–0.0)	100.0 (100.0–100.0)	< 0.001	7.4 (6.9–8.4)	-^a^	**Absence	0.004
	*E. coli*	43.9 (34.1–46.1)	0.0 (0.0–0.0)	100.0 (100.0–100.0)	<0.001	7.2 (6.9–7.34)	-^a^	**Absence	0.002
	*S. aureus*	32.7 (23.7–49.7)	0.0 (0.0–0.0)	100.0 (100.0–100.0)	0.008	7.6 (7.4–7.8)	-^a^	**Absence	0.002

P values were obtained using Mann–Whitney U test

*MO* microorganism, *IQR* interquartile range, *cfu* colony forming units, *C + * positive control, *ALT* antibiotic lock therapy

^a^No cells were recovered after therapy. Cfu/ml resulted to be 0 (0.0-0.0) for all microorganisms after every therapy being log_10_ of 0 in-calculated

^b^No p75 value obtained

^*^Limit of detection of cfu counting using conventional culture was 10 cfu/ml

**In prophylaxis therapy, no reduction could be measured, as there was no pre-formed biofilm. Results are expressed as absence

Reduction in live cells and cfu counts was statistically significant in both therapies for all microorganisms. Moreover, this reduction reached 100% for all microorganisms in both therapies except for live cells of *P. aeruginosa* and *E. coli*.

### Treatment therapy (ALT)

**Fig. 3:**
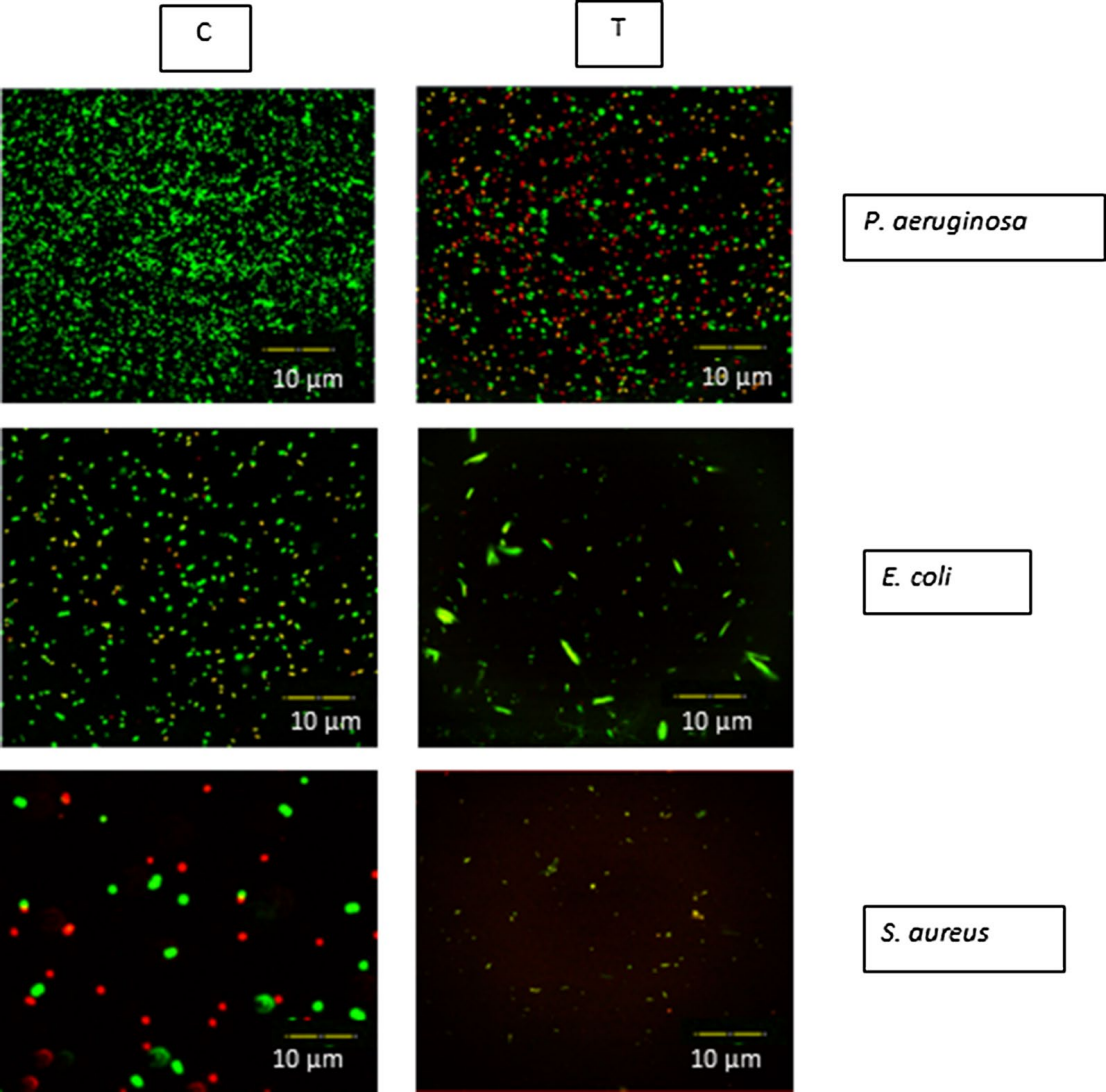
Live cells of the different bacterial biofilm measured by CLSM after a treatment therapy with a selective digestive decontamination solution. *C* positive control; *T* treated sample

**Fig. 4:**
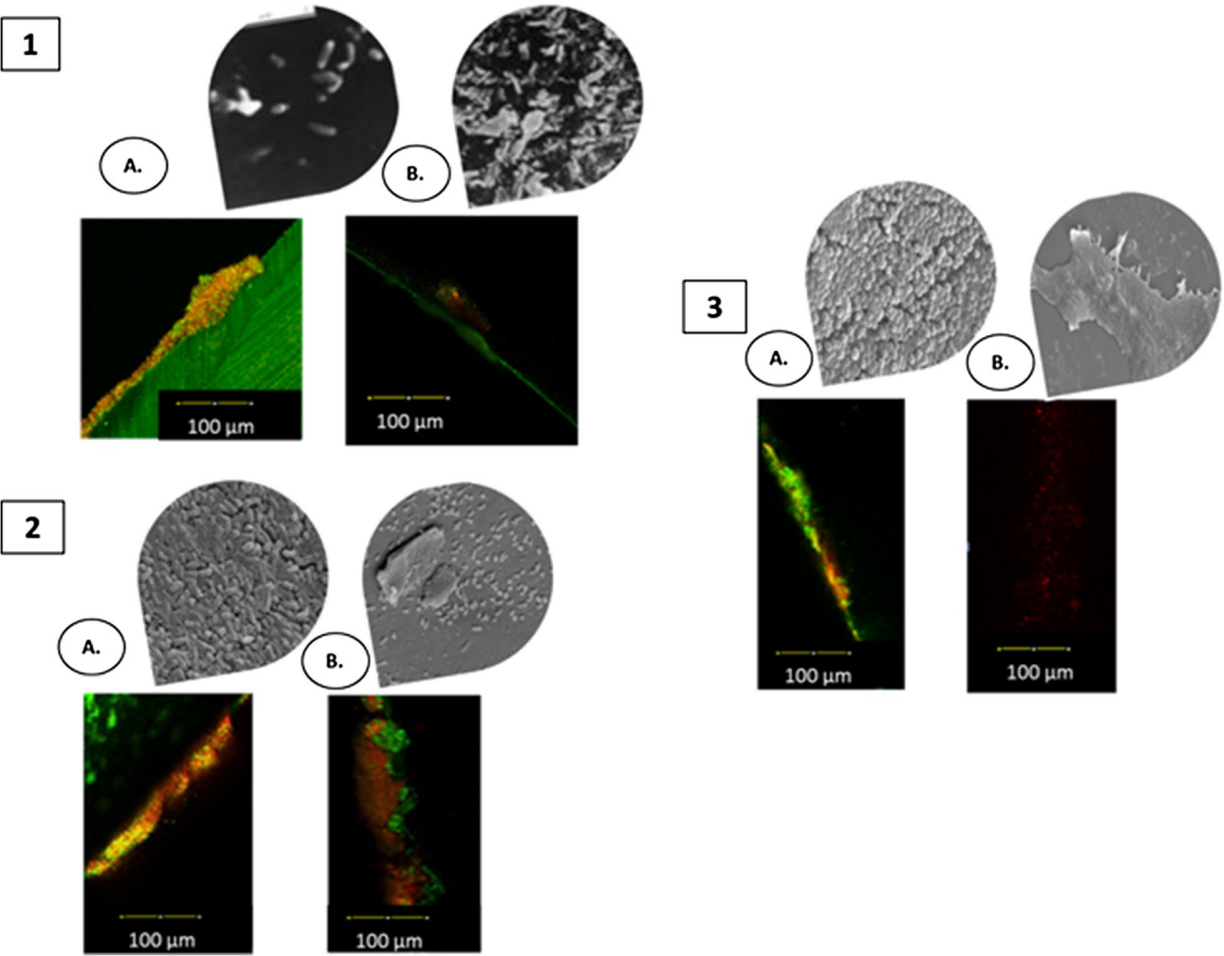
CLSM and SEM images magnified at 3000X of bacterial biofilms after treatment therapy with SDDs. 1 Pseudomonas aeruginosa; 2 Escherichia coli; 3 Staphylococcus aureus; *A* positive control; *B* treated sample

Median (IQR) percentage reduction of live cells of *P. aeruginosa, E. coli,* and *S. aureus* treated samples were, respectively (Table 1): 53.2% (39.4%—64.1%), 67.9% (46.7%–78.7%), and 100% (100%–100%), which corresponded to a statistical significant reduction on the percentage of live cells between treated and non-treated samples (p < 0.001, p = 0.002, and p = 0.007, respectively) (Fig. 3). Percentage reduction of cfu/ml was 100% for *P. aeruginosa*, *E. coli,* and *S. aureus* treated samples (p = 0.002) (Table 1). Total number of cfu/ml are collected in supplementary material (Additonal file 1: Table S1). This represented that non-culturable cells were collected either from *P. aeruginosa*, *E. coli,* or *S. aureus* biofilms. Thickness and biofilm structure visualized by CLSM and SEM are shown in Fig. 4. Positive controls are characterized by a thickness layer of cells embedded into an extracellular matrix (ECM) whereas in treated samples, the ECM was disrupted, and abnormalities in cell size and shape were observed.

### Prophylactic therapy (ALP)

**Fig. 5:**
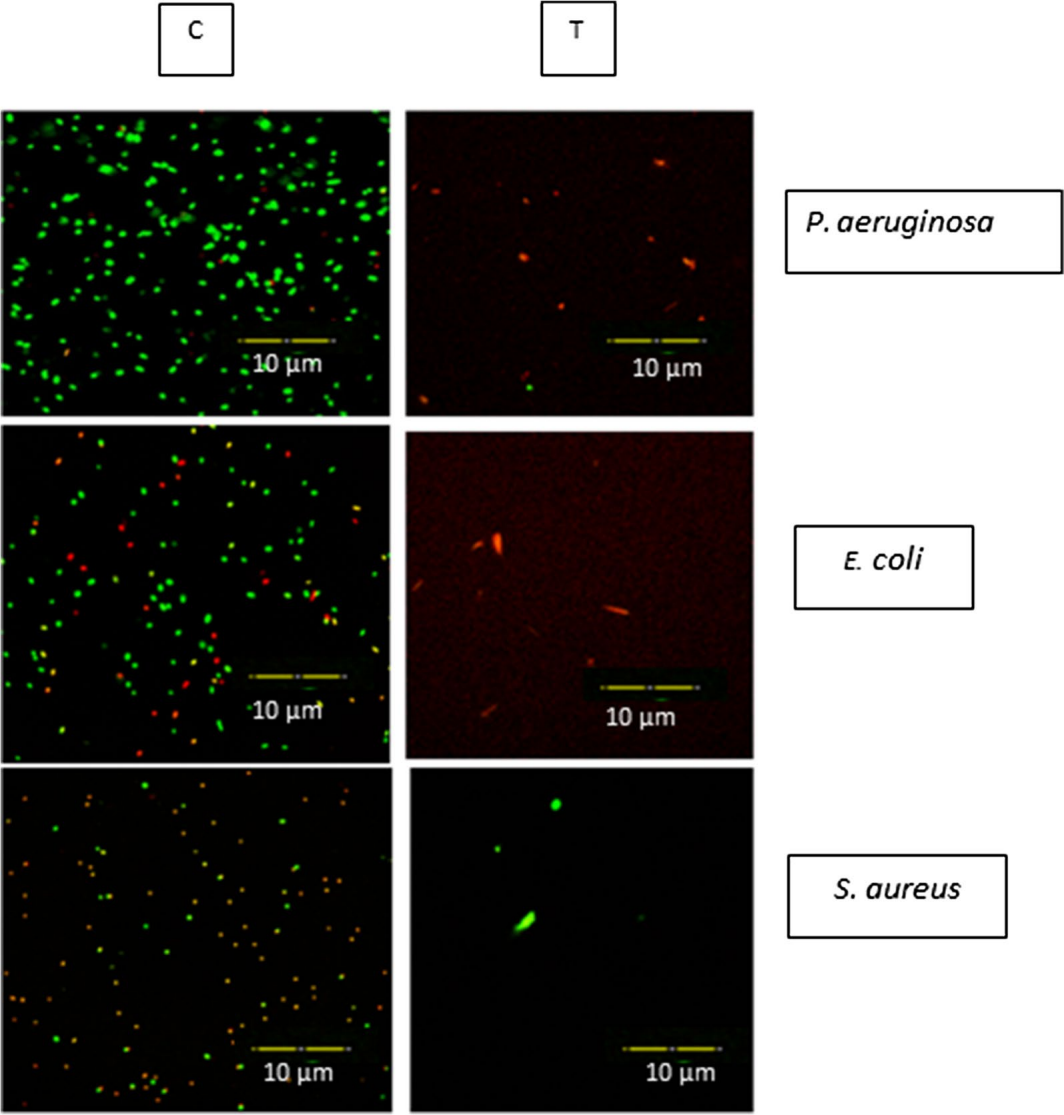
Live cells of the different bacterial biofilm measured by CLSM after a prophylactic therapy with a selective digestive decontamination solution. *C* positive control; *T* treated sample

**Fig. 6:**
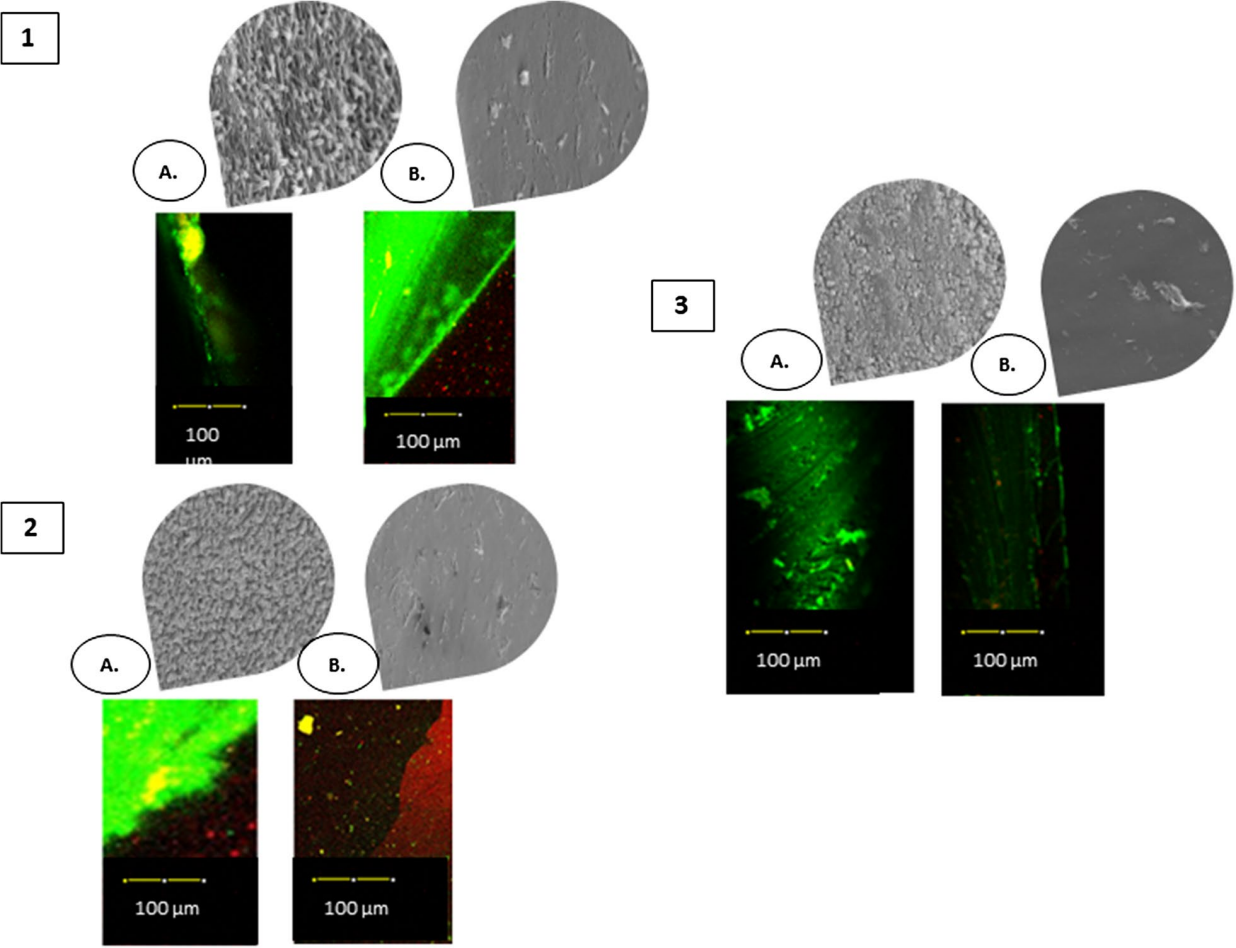
CLSM and SEM images magnified at 3000X of bacterial biofilms after prophylactic therapy with SDDs. 1 Pseudomonas aeruginosa; 2 Escherichia coli; 3 Staphylococcus aureus; *A* positive control; *B* treated sample

In the ALP, there was absence of live cells of *P. aeruginosa*, *E. coli,* and *S. aureus* treated samples, p < 0.001, p < 0.001, and p = 0.008, respectively (Table 1 and Fig. 5). Besides, there was also absence of cfu/ml counts for the 3 microorganisms (limit of detection < 10 cfu/ml) (p = 0.004 for *P. aeruginosa* and p = 0.002 for *E. coli* and *S. aureus*). Total number of cfu/ml are collected in Additional file (Additional file 1: Table S1). Figure 6 illustrates changes in thickness and biofilm structure after prophylactic therapy with SDDs compared with positive controls when visualized with CLSM and SEM. Using SEM we observed how ECM disappeared in treated samples, making bacteria vulnerable to antibiotics, and only antibiotic crystals and cell debris were presented on ETT surface after the therapy.

### Filtration testing

In the bench-top model the Falcon tube represented the trachea of the patient. We did not recovered any volume of SDDs or saline in the tube after finishing the ALT and ALP therapies. Hence, all the treatments were retained in the cuff of the ETT.

## Discussion

Ventilator-associated pneumonia still represents a challenge in ICU patients with an incidence up to 24%, an attributable mortality of 13% and an increase of hospital costs of around US$55,882 per patient [33, 34]. A wide variety of preventing measures have been proposed by the Society for Health Epidemiology of America (SHEA) and IDSA including daily oral care, semi-recumbent position, subglottic secretion aspiration, and SDD [19, 34–36]. Most of these measures are based mainly on the decolonization of the internal surface of the ETTs. However, it has been demonstrated that bacterial biofilm can also be formed on the external surface of ETTs [37].

Under this basis, we conducted an in vitro study using a bench-top model of adult trachea intubation where we have demonstrated that SDD can be applied as lock therapy for the prevention of biofilm formation in the external surface of the subglottic space of ETT with an efficacy of 100% for *P. aeruginosa* ATCC 15442, *E.coli* ATCC 25922, and *S. aureus* ATCC 29213. Based on the possibility to prevent VAP, other authors have demonstrated other procedures to prevent biofilm formation. Machado et al., have recently described a novel way of preventing biofilm formation on ETT surface by nanomodifying polyvinyl with a fungal lipase [38, 39]. They also demonstrated that these modifications reduced *P. aeruginosa* colonization by 2.7 log_10_ [40]. However, no total reduction was observed. In our study, no live or cultivable cells appeared after the prophylactic therapy with SDDs. Another study conducted by Wang et al., an inhibition of *P. aeruginosa* biofilm formation was achieved using ultrasonic guided waves on a new model of ETT [41]. Although ultrasonic guided waves are a promising technology, it is still more cost effective than SDDs. Furthermore, we have previously demonstrated that promising results were obtained for *P. aeruginosa* biofilm reduction using SDDs as treatment therapy comparing single-dose and 5-day locking therapy in our model [42]. Thus, more studies are needed to test its efficacy against other microorganisms and to prove the application as a preventive measure.

*P. aeruginosa*, *S. aureus,* and *E. coli* are the three main representative microorganisms of VAP with an incidence of 13.1%, 28.3%, and 6%, respectively [43]. Literature about prevention of *P. aeruginosa* colonization in ETT is wide spread but ours is the first study in which prophylactic therapy has been performed in three different microorganisms with successful results for all of them.

Another key point in the management of VAP is the treatment. Although preventing measures have been described of being efficient, bacteria biofilm is still difficult to eradicate from ETT surface due to chronicity of infections and treatment failure [9]. We noted that SDDs reduced a pre-formed biofilm of *P. aeruginosa*, *E. coli,* and *S. aureus*. Although percentage of live cell reduction did not achieve 75% in *P. aeruginosa* and *E. coli*, it could be used as supportive treatment for actual therapies. In contrast, when measured by cfu counts, the percentage of reduction is 100%. We hypothesize that this issue is explained by the viable but non-culturable (VBNC) cell phenomena. It is characterized by the ability of bacteria of reducing their metabolic activity and change their membranes and walls to survive under unfavorable conditions such as starvation or high stress [44]. During the 5 day ALT, bacteria are exposed to high doses of antibiotic leading to an adaptive response in a try of survival making diagnose and treatment by classical methods more challenging. As commented in the study of Li et al., the role of VBNC cells is still under discussion. Some authors support the hypothesis that these cells are in a preliminary phase of dying and is believed to have no clinical impact. Other authors consider that VBNC cells would have the ability to revive. However, in the prophylactic group of our study, neither cfu nor viable cells were recovered from any of the microorganisms tested. This means that VBNC cells were not present and therefore it would not have a negative impact in a clinical scenario.

In *S. aureus*, we have observed that SDDs supplemented with vancomycin eradicated bacteria biofilm in a 100%. In a study presented by Fernandez-Barat et al., they evaluated the capacity of vancomycin versus linezolid to eradicate *S. aureus* methicillin-resistant biofilm on ETT surface o ventilated pigs [32]. They observed that i.v. linezolid was statistically significant better than vancomycin but no eradication was obtained. Thus, the administration of linezolid plus ALT with SDDs could achieve better results in vivo for VAP treatment. Although VBNC was not observed in *S. aureus* model, vancomycin and daptomycin have shown to induce significant rates of viable but non-culturable *S. aureus* cells [45, 46].

Regarding the clinical application of this procedure, Penumatikos et al. have previously demonstrated in a clinical study that VAP was significantly reduced when continuous infusion of antibiotic solution was applied at the subglottic space in trauma patients [29]. However, they administered the antibiotic solution by continuous infusion, which seems more difficult to manage in ICU patients. We consider a more easy-to-use regimen with a 30 min-lock therapy applied per nursing shift (every 6 h) just immediately before subglottic aspiration. However, as we only demonstrated that there was no evidence of leaking past the cuff of the ETT in the in vitro model, it is necessary to validate this finding in a real clinical scenario to assess that this regimen would be a safety procedure to be performed in intubated critical patients. We consider that when a “lock therapy” at the subglottic space is aimed it may not cause damage to the patient because of SDDs filtration to the lungs mainly because of the continuous cuff pressure monitoring > 20 cm H_2_O, as recommended by the guidelines [18].

Although our in vitro bench-top model mimics the ETT subglottic conditions during patient-use of this device, it was a static model that may not simulate the real scenario in an intubated patient. Moreover, SDD could not reach the distal ETT and the cuff, however, as SDD is also applied by gastrointestinal and oral sources, it could reach other parts than the subglottic space. Methodologically, we have not used any neutralizer to inactivate the antibiotics before plating which may limit the VBNC bacteria growth although there is still no consensus about neutralizers and VBNC cells. Besides, guidelines do not contemplate its use in clinical samples except for decontamination before processing samples for mycobacteria cultures [44, 47, 48]. Another limitation of the study was the small amount of microorganisms used. We used the three main representative microorganisms to cause VAP in our institution. However, these results may not be extrapolated to others institutions where *P. aeruginosa, E. coli* and *S. aureus* represent low rates of VAP, and hence, further studies should be perform with other etiological pathogens. Our results must be validated in the real clinical practice by randomized clinical trials.

## Conclusion

Ours is the first study to demonstrate that SDDs used as lock therapy in the subglottic space can represent an additional successful prophylactic measure against the biofilm of three of the most common microorganisms causing VAP. This means that it could be used in combination with traditional oral and digestive decolonization procedure, as it also demonstrated not to cause antibiotic resistance [26, 27, 49–51]. Further clinical investigations should be performed to evaluate its efficacy and safety in clinical settings.

## Supplementary information

**Additional file 1: Table S1.** Cfu/ml counts for *P. aeruginosa*, *E. coli* and *S. aureus* after treatment and prophylactic therapies with SDD lock solution. **Figure S1.** Flowchart of the procedure for prophylaxis and treatment therapy of SDD lock solution in ETT.

## Data Availability

Data sharing is not applicable to this article as no datasets were generated or analysed during the current study.

## Abbreviations

TSB: Tryptic soy broth
BHI: Brain heart infusion
LB: Luria broth
PBS: Phosphate buffer solution
