# Supplementary material for: Selective digestive decontamination solution used as “lock therapy” prevents and eradicates bacterial biofilm in an in vitro bench-top model
Source: Ann Clin Microbiol Antimicrob. 2020 Sep 23;19:44. doi: 10.1186/s12941-020-00387-7 (PMC7513905; doi:10.1186/s12941-020-00387-7)
Supplement: Supplementary file 1 — Additional file 1: Table S1. Cfu/ml counts for P. aeruginosa, E. coli and S. aureus after treatment and prophylactic therapies with SDD lock solution. Figure S1. Flowchart of the procedure for prophylaxis and treatment therapy of SDD lock solution in ETT. [file 12941_2020_387_MOESM1_ESM.docx]

**Additional file 1**

**Table S1. Cfu/ml counts for *P. aeruginosa*, *E. coli* and *S. aureus* after treatment and prophylactic therapies with SDD lock solution.**

| **Microorganism** | **Therapy** | **Solution** | **Median (IQR) cfu/ml** |
| --- | --- | --- | --- |
| *P. aeruginosa* | Prophylaxis | ALT | 0 (0-0)* |
|  |  | SLT | 2.3x10^7^ (8.6x10^6^-7.8x10^7^) |
|  | Treatment | ALT | 0 (0-0)* |
|  |  | SLT | 3.8x10^7^ (2.3x10^7^-2.5x10^8^) |
| *E. coli* | Prophylaxis | ALT | 0 (0-0)* |
|  |  | SLT | 1.4x10^7^ (5.2x10^6^-2.6x10^7^) |
|  | Treatment | ALT | 0 (0-0)* |
|  |  | SLT | 1.2x10^7^ (4.9x10^6^-2.7x10^7^) |
| *S. aureus* | Prophylaxis | ALT | 0 (0-0)* |
|  |  | SLT | 3.9x10^7^ (2.4x10^7^-9x10^7^) |
|  | Treatment | ALT | 0 (0-0)* |
|  |  | SLT | 4.9x10^7^ (2.7x10^7^- 7.6x10^7^) |

**SDD**, selective digestive decontamination; **ALT,** antibiotic lock therapy; **SLT,** saline lock therapy; **IQR**, interquartile range; **cfu**, colony forming units.

*Limit of detection of cfu counts using conventional culture was 10 cfu/ml.

**Figure S1. Flowchart of the procedure for prophylaxis and treatment therapy of SDD lock solution in ETT.
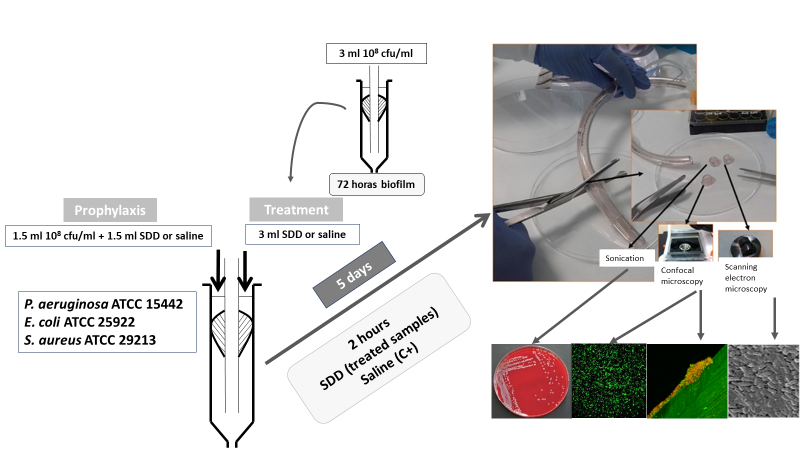
**

**72 hours biofilm**

**SDD**, selective digestive decontamination; **ETT**, endotracheal tube; **cfu**, colony forming units; **C+**, positive control.
